# Pro re nata prescribing and administration for neuropsychiatric symptoms and pain in long-term care residents with dementia and memory problems: a cross-sectional study

**DOI:** 10.1007/s11096-019-00883-7

**Published:** 2019-07-24

**Authors:** Alys W. Griffiths, Claire A. Surr, David P. Alldred, John Baker, Ruchi Higham, Karen Spilsbury, Carl A. Thompson

**Affiliations:** 1 Centre for Dementia Research, School of Health and Community Studies, Leeds Beckett University, City Campus, Leeds, LS1 3HE UK; 2 School of Healthcare, Faculty of Medicine and Health, University of Leeds, Leeds, LS2 9JT UK

**Keywords:** Analgesic, Antipsychotics, Dementia, Long-term care, Neuropsychiatric symptoms, Pain, Pro re nata, United Kingdom

## Abstract

*Background* Prescribing, dispensing and administering pro re nata (PRN; as needed or necessary, as determined by an individual) medicines to people with intermittent or short-term conditions is a potential area for medication errors and inappropriate prescribing and administration. In people with dementia, regular PRN medicines use can demonstrate good practice when appropriate or poor in situations where their use is not recommended. However, the frequency of PRN prescription and administration within long-term care settings (care homes) for people with dementia is largely unknown. A limited number of studies worldwide suggest variation between countries. *Objective* To describe the prescription and administration rates of PRN medicines for people with dementia in UK care homes. *Setting* Fifty UK care homes. *Method* Medication details were collected from review of residents’ medicines records within the care home for the previous month. *Main outcome measure* Prescription and administration of PRN medicines for the treatment of behaviours associated with neuropsychiatric symptoms and pain. *Results* The most commonly prescribed PRN medicines were analgesics (35.3%), although lower levels of PRN prescription were observed compared to recent studies. The percentage of residents receiving PRN administrations varied, with 20% for antipsychotics, 50% for benzodiazepines, 59% for analgesics, and 85.7% for nonbenzodiazepine hypnotics being administered. *Conclusion* Further research is needed to understand the decision making in PRN prescription and administration within long-term care. The prescribing of potentially inappropriate medicines remains a problem in long-term care settings and pharmacists have a key role in reducing inappropriate polypharmacy by undertaking medication reviews that consider both regular and PRN medicines.

## Impact on practice

- There are inconsistencies in practice for people living with dementia and memory problems in care homes, and regular medication reviews are required.
- Non-pharmacological alternatives should be considered for the management of neuropsychological symptoms for people living with dementia and memory problems.
- Particular attention should be given to ensure appropriate antipsychotic prescription for people with dementia and memory problems in care homes. Antipsychotics should only be used where absolutely necessary, and only in the short term.

## Introduction

Dementia affects approximately 47 million people worldwide, and this is projected to rise [1]. Around 20–40% of people with dementia live in long-term care [2, 3]. Multimorbidity (having more than one long term condition) [4] and thus polypharmacy (taking multiple medicines; often defined as ≥ 4 medicines [5]) is highly prevalent and concerning in long-term care, with 91% of long-term care residents taking 5+ medicines [5]. A study conducted in England suggested that risks of polypharmacy include multiple side effects, increased risk of preventable hospitalisations, impaired quality of life, increased likelihood of interactions between medicines [4] and higher risks of medication errors, which are common in this population [6, 7]. In addition, inappropriate (over or under) prescribing, is commonplace [8] with implications for health and well-being and associated cost burden to the healthcare system. On average, 50% (range 24–80%) of people living in long-term care receive at least one inappropriate medicine and up to 30% of residents with advanced dementia are prescribed medicines classified as ‘never appropriate’ for this population [9]. Therefore, understanding the prescription and administration of medicines for residents in long-term care is a clinical and policy imperative.

### PRN medicines in long-term care

Prescribing, dispensing and administering *pro re nata* (PRN; as needed or necessary, as determined by an individual) medicines to people with intermittent or short-term conditions is a potential area for medication errors and inappropriate prescribing/administration. PRN medicines are given when an individual requires them, rather than as a regular daily dose or at specific times e.g. during a medication round [10]. Long-term care residents with dementia who may have impaired communication rely on staff making accurate and timely judgements of need for administration of PRN medicines. For example, those with impaired communication or who have difficulties taking medicines have been noted to receive less PRN medicines [11]. As a result, guidelines have been developed aimed at improving appropriate assessment and administration of PRN medicines in long-term care settings [12, 13].

Alongside warranted variations in PRN administration between residents, unwarranted variations between long-term care settings exist, suggesting PRN use may not be based on individual resident needs, but organisation level factors, such as staffing levels and care home size [11, 14] and staff confidence or knowledge to make judgments about the necessity for PRN medicine administration [15]. The absence of clear instructions for staff on how and when to administer medicines increases the likelihood of adverse events or errors [12]. Therefore, PRN medicines administration may be a signal of underlying care culture and quality and a risk factor in poorer quality care settings.

In people with dementia, regular PRN medicines use can demonstrate good practice when appropriate (e.g. PRN administration of appropriate analgesics may indicate effective assessment and management of acute or intermittent pain) or poor practice (e.g. PRN administration of antipsychotics may indicate use as a first rather than last resort for managing agitation). This is of concern given the increased risks of these drugs for falls, stroke and death [16] and their recommendation for use only in the short term, if closely monitored and after all psychosocial approaches have been exhausted [17]. Frequent administration of PRN medicines such as pain relief however, may indicate the need for a medication review as a PRN prescription may be inappropriate. For example, antipsychotics should be limited to residents who are severely distressed or at risk of harming themselves or others, and where non-pharmacological interventions have failed. Whilst antipsychotics have proven modest efficacy, side effects can be disabling and they increase the risk of cognitive decline, stroke and death [18]. Likewise the use of certain non-benzodiazepine hypnotics known as Z drugs has been found to increase of bone fractures and death [19]. Therefore prescription and administration of medicines such as anti-psychotics, benzodiazepines, non-benzodiazepines, hypnotics and analgesia to people with dementia are of particular interest as they present potential high risks for well-being, quality of life and safety if administered incorrectly or not according to need.

However, the frequency of PRN prescription and administration within long-term care settings (care homes) is largely unknown. A limited number of studies worldwide suggest variation between countries. PRN prescription rates in care homes range from 91% in the USA [20] and 75% in Germany [21] to just 31% in Norway [22]. Significant within-country variations also exist: three studies conducted in Australian long-term care settings found overall PRN prescription rates of 94% [23], 84% [11] and 7% [24]. Other studies have examined PRN medicine prescribing on a specific drug type basis. For example, the frequency of antipsychotic PRN prescription was 24.5% in a Canadian study [25], 23% in an Australian study [26], and 37% [27] and 15% [28] in two German studies. Analgesic prescribing on a PRN basis ranges from 40% in Germany [29], 48% in the USA [30], and 72% in Norway [31]. Where reported, the most commonly prescribed PRN medicines are analgesics [20, 21, 23, 32, 33] or anxiolytics/hypnotics [24].

Administration levels also vary; one US study reported administration rates of 30% over 7 days, while two Australian studies reported rates of 54% [11] and 28% [23]. Individual medication-type PRN administrations also varied: administrations of analgesia were 30% in the USA [20] but only 12% in Norway [31]. Antipsychotic PRN administration rates are generally low. A Canadian study reported only 2.1 PRN administrations on average per resident per month, with most residents receiving no administrations [25]. In an Australian study, 11% of residents received at least one administration in a month [26], whilst in Germany this figure was just 6% [28].

Within the existing literature, two studies were specifically conducted with people living with dementia [20, 24] and dementia prevalence within the remaining samples ranged from 54 to 98.5% [26, 31]. Where this was examined those with a PRN prescription were more likely to have dementia ([28], 61 vs 39% [34]). However, one study [33] found no difference in the likelihood of PRN prescription in residents with and without dementia. Therefore, little is known about the prescribing or administration of PRN medicines to people living with dementia in UK care homes.

In the present study, we were interested in the clinically significant class of PRN medicines related to the management of neuropsychiatric symptoms and pain, which if not managed can lead to neuropsychiatric symptoms [35]. Pain assessment and management for people with dementia is frequently suboptimal [29]. Despite known risks, and evidence suggesting widespread prescription but variable degrees of administration of PRN medicines, there is very limited understanding of PRN medication use in care homes for people living with dementia [36].

## Aim of the study

We aimed to provide the first reported data on the rates of prescription and administration of PRN medicines associated with neuropsychiatric symptoms and pain management for people living with dementia and memory problems in UK long-term care settings (care homes).

## Ethics approval

Ethical approval for the DCM-EPIC trial was gained from the Yorkshire and the Humber Leeds Bradford NHS REC [reference number 13/YH/0016]. Consent from study participants included permission to reanalyse data for research purposes. Ethical approval for this sub-study was granted by Leeds Beckett University Research Ethics Committee.

## Method

Data analysed in this paper was collected as part of the DCM-EPIC trial, a UK randomised controlled trial in care homes. The trial aimed to establish the effectiveness of a person-centred intervention for the quality of life of people living with dementia or memory problems. Baseline data (collected from May 2014–December 2015) on prescribed and administered medication were analysed for this paper.

### Participants

Participants were recruited from 50 care homes in the United Kingdom. Residents with dementia diagnoses or memory problems (i.e. without a confirmed dementia diagnosis) were eligible to participate. Residents formally admitted to an end of life care pathway or who only resided semi-permanently were ineligible.

### Procedure

Care homes were selected by listing all potentially eligible care homes across the three recruitment areas (West Yorkshire, Oxfordshire and South London) using publicly available data. They were then randomly ordered within post-code area/boroughs and the first 10–12 listed care homes approached from one post-code/borough in each hub. This method rotated across each postcode/borough until sufficient homes were recruited. Approach was initially made by post, followed by a researcher phone call. Eligibility was confirmed for any homes potentially interested in participation ahead of consent. Care homes were recruited randomly, stratified by postcode, size, and type. Homes subject to regulatory enforcement or who were deemed ‘inadequate’ by the UK quality regulator (the Care Quality Commission) were ineligible to participate.

Medication details were collected from residents’ medicine administration records for the previous month, held within their care home. Prescription of medicines relevant to the management of neuropsychiatric symptoms associated with dementia, physical and psychological health problems (e.g. antipsychotic, benzodiazepine, mood stabiliser) and pain (analgesic) and frequency of administration of these if prescribed on a PRN basis, was recorded on a standardised form by researchers. Demographic data including comorbidities was collected from resident care records.

Additionally, information about symptoms of agitation was collected using the Cohen-Mansfield Agitation Inventory (CMAI [37]), which measures 29 behaviours typically associated with agitation/aggression. Staff members identified the frequency of 29 behaviours during the previous 2 weeks, rated on a seven-point Likert scale (1–7) ranging from “never” to “several times an hour”. This measure shows moderate concurrent validity with scores on the Neuropsychiatric Inventory—Nursing Home version Agitation sub-scale (*r *= .52 [38]).

### Statistical analysis

We calculated descriptive data (counts and means) by resident and care home. To explore our hypothesis that individual care home approach to medicines use were related to rates of PRN administration, we calculated Pearson’s correlation coefficients using SPSS version 25.

## Results

### Demographic characteristics

A total of 728 participants were recruited from 50 care homes (31 residential or nursing home, 19 dementia care specialist home). Care homes provided care for an average of 31.8 residents, with an average of 77.7% of residents having dementia (see Table 1 for participant demographics). Participants had, on average, two comorbidities alongside dementia (range 0–14). Selected comorbidities often associated with PRN prescriptions included depression (n = 117, 16.1%), anxiety (n = 57, 7.9%), psychosis (n = 40, 5.5%), sleep disturbance (n = 13, 1.8%), and delirium (n = 5, .7%). Demographic data were missing for two participants who were not included in analyses, providing a total of 726 participants.

**Table 1:** Participant demographics

*Characteristics*	
Age at registration (years) M (SD)	85.6 (7.64)
*Gender*	
Female	536 (73.8%)
Male	190 (26.2%)
Length of stay in care home (years) M (SD)	2.3 (2.34)
*Ethnicity*	
White British/European	702 (96.7%)
Other	24 (3.3%)
*Funding type*	
Local authority	352 (48.5%)
Self-funded	289 (39.8%)
Local authority and self-funded	34 (4.7%)
Continuing healthcare	48 (6.6%)
Missing	3 (.4%)
*Dementia severity (measured by functional assessment staging tool)*	
1–3	6 (1%)
4	95 (13.6%)
5	74 (10.6%)
6	380 (54.5%)
7	142 (20.4%)
Missing	29 (3.9%)

### Medication use

The total number of regular medicines prescribed per resident ranged from 0 to 28 (mean = 8.68, SD = 4.14) with 0–3 on a PRN basis (Mean = .4, SD = .60). Eight residents (.1%) were not prescribed any medicines. The total number of medication prescriptions was 6266, of these 5949 (95.0%) were regularly prescribed and 317 (5.0%) prescribed on a PRN basis. The prevalence of regularly and PRN prescribed medicines is shown in Table 2, stratified by gender, age and regular medication prescription, in line with previous reporting [21, 22].

**Table 2:** Pro re nata (PRN) prescribing for residents in care homes stratified by demographic characteristics (N = 726)

	No PRN medicine	1 PRN medicine	2 PRN medicines	3 PRN medicines
*Age*				
< 70	21 (70)	7 (23)	2 (7)	–
70–79	86 (64)	37 (28)	9 (7)	2 (1)
80–89	232 (67)	93 (27)	19 (5)	4 (1)
≥ 90	130 (61)	68 (32)	14 (7)	2 (< 1)
All	469 (65)	205 (28)	44 (6)	8 (1)
*Gender*				
Female	332 (62)	182 (34)	20 (4)	2 (< 1)
Male	137 (72)	44 (23)	6 (3)	3 (2)
*Regular prescriptions*				
M (SD) range	8.03 (4.11) 0–28	8.72 (3.92) 1–23	8.81 (3.69) 3–17	8.60 (5.08) 4–16
0–4	92 (69)	37 (29)	2 (1)	2 (1)
5–9	230 (66)	102 (30)	14 (4)	1 (< 1)
≥ 10	147 (60)	87 (35)	10 (4)	2 (1)

All numbers provided as N (% of category) unless otherwise specified

The most frequently prescribed PRN medication were analgesics with over a third of participants (35.3%) prescribed them (see Table 3 for breakdown of type of medication prescribed). All other PRN prescriptions were at very low levels, ranging from .4% for mood stabilisers to 5.4% for benzodiazepines. The class/individual medicines prescribed and administered can be seen in Table 4.

**Table 3:** Prevalence of prescribed and administered regular and PRN medicines (N = 726)

Medication	N residents (%) prescribed	N medicines prescribed	N (%) medicines administered of prescribed	Number of administrations per resident of those administered at least once over a month [M (SD) range]
*Antipsychotic*				
Regular	93 (12.8)	96	–	–
PRN	10 (1.4)	10	2 (20)	4.14 (8.30) 0–22
*Benzodiazepine*				
Regular	43 (6.0)	47	–	–
PRN	38 (5.4)	39	19 (50)	18.62 (35.84) 0–137
*Non-benzodiazepine hypnotic*				
Regular	40 (5.5)	40	–	–
PRN	6 (1.0)	6	5 (85.7)	28.83 (47.30) 0–123
*Antidepressant*				
Regular	268 (37.0)	299	–	–
PRN	3 (.4)	3	3 (100)	31 (41.87) 2–79
*Cognition enhancing medicines*				
Regular	171 (23.6)	177	–	–
Anticonvulsants				
Regular	35 (4.8)	40	–	–
*Mood stabilisers*				
Regular	3 (.4)	3	–	–
*Analgesic*				
Regular	356 (50.1)	468	–	–
PRN	256 (35.3)	259	151 (58.3)	25.14 (36.43) 0–122

**Table 4:** Medicines prescribed by name and type

Medication	Total medicines prescribed	Regular	PRN
Antipsychotic	106	96	10
Amisulpride	6	5	1
Aripriprazole	5	5	–
Chloropromazine	1	1	–
Flupentixol	3	3	–
Haloperidol	4	4	3
Levomepromazine	2	–	2
Olanzapine	17	17	–
Prochlorperazine	2	1	1
Quetiapine	19	19	–
Risperidone	43	40	2
Benzodiazepine	86	47	39
Clonazepam	2	2	–
Diazepam	24	13	11
Lorazepam	46	19	27
Midazolam	1	1	–
Nitrazepam	4	4	–
Oxazepam	1	1	–
Temazepam	8	7	1
Non-benzodiazepine hypnotic	46	40	6
Chlormethiazole	1	–	1
Melatonin	1	1	–
Zopiclone	44	39	5
Antidepressant	302	299	3
Amitriptyline	18	18	–
Citalopram	88	88	–
Dosulepin	1	1	–
Duloxetine	2	2	–
Escitalopram	2	2	–
Fluoxetine	9	9	–
Lofepramine	3	3	–
Mirtazapine	80	80	–
Paroxetine	2	2	–
Sertraline	26	26	–
Trazodone	61	58	3
Venlafaxine	7	7	–
Cognition enhancing medicines	177	177	–
Donepezil	87	87	–
Galantamine	19	19	–
Memantine	55	55	–
Rivastigmine	16	16	–
Anticonvulsants	40	40	–
Carbamazepine	10	10	–
Gabapentin	2	2	–
Lamotrigine	1	1	–
Levetiracetam	7	7	–
Phenobarbital	3	3	–
Phenytoin	2	2	–
Pregabalin	1	1	–
Sodium valproate	12	12	–
Topiramate	1	1	–
Mood stablisers	3	3	–
Lithium	3	3	–
Analgesic	724	468	256
Aspirin	102	101	1
Buprenorphine	21	21	–
Co-codamol	55	34	21
Codeine	42	27	15
Ibuprofen	23	18	5
Morphine	13	6	7
Paracetamol	447	243	204
Tramadol	9	8	1
Other	12	10	2

### Correlation analyses

Neither the total number of prescribed medicines (*r* = .03, *p *> .05), nor the number of PRN medicines administered (*r* = .14, *p *> .05) correlated with care home.

Level of agitation (as measured by the CMAI), was associated with total number of PRN prescriptions (*r *= .08, *p *< .05), but not total number of regular and PRN prescriptions (*r *= − .06, *p *> .05).

## Discussion

The current study presented the first evidence on PRN medication prescribing and administration within UK long-term care settings (care homes) for people with dementia. The most commonly prescribed PRN medicines were analgesics, in line with most recent evidence [21]. However, this may not be universally positive as many staff members lack the skills to perform adequate pain assessments, meaning residents do not always receive analgesic medicines when needed [30]. This is particularly important as cognitive impairment is associated with decreased likelihood of receiving PRN analgesics [31]. In addition, continuing potentially inappropriate long-term PRN analgesics without review, for example nonsteroidal anti-inflammatory drugs (NSAIDs), may mask or cause potentially harmful interactions or adverse effects [39, 40]. Regular and PRN NSAIDs can cause peptic ulcer disease, may increase thrombotic risk and impair renal function with the potential for Acute Kidney Injury. NSAIDs also interact with many commonly prescribed medicines, including antihypertensives, antithrombotics, lithium and Selective Serotonin Reuptake Inhibitors [41]. Unfortunately, we did not collect data on the underlying reasons why medications were prescribed (see study limitations). We therefore cannot rule out the possibility that staff behaviours relating to PRN use for people with dementia increase exposure to potentially inappropriate medications with an unfavourable balance of benefits and harms, when compared to alternative treatment options. More research is needed to ascertain the degree to which a diagnosis of dementia modifies the probability of meeting STOPP (Screening Tool of Older Person’s Prescriptions) or Beers criteria or not benefiting from START (Screening Tool to Alert doctors to Right Treatment) criteria [39, 40].

Our data was collected after publication of guidance on antipsychotic medication prescribing for people with dementia within the UK [12]. This suggested antipsychotic medication should be used as a last resort, and not longer than 6 weeks at a time. Within our sample, 1.4% had a prescription for an antipsychotic medication on a PRN basis and 12.7% on a regular basis. However, we cannot know whether the levels observed are specific to our sample without drawing comparisons to other care home samples within the UK. Only one study has considered both regular and PRN antipsychotic use, where Managers reported that 8% of residents had a regular and 4% of residents had a PRN antipsychotic prescription [42], however caution should be exercised in interpreting this self-reported data from managers. The study also did not report how frequently prescribed PRN medications were administered. As our data were cross-sectional we do not know how long regular antipsychotic medicines had been prescribed. A low level of PRN antipsychotic prescriptions was reported in a recent Australian study [23], although their seven-day data collection period included only one administration on average, whereas within our sample, there were two administrations across 1 month. This may reflect recent increased understanding about the potential harms of antipsychotic administration for people living with dementia [23] or the presence of good practice guidelines for care homes to work with [12, 13]. Whilst we observed generally low levels of PRN prescriptions and administrations, it is not known whether this is in response to the guidelines, as no evidence exists for the prevalence of PRN prescriptions in the UK before their development.

PRN medication administration varied within the present study. For some categories, such as antipsychotics, only 2 of 10 residents received an administration within the one-month data collection period. Other categories, such as benzodiazepines and non-benzodiazepine hypnotics, were far more common. For example, 50% of participants prescribed these on a PRN basis received at least one administration during the month. However, some residents received over 100 administrations of benzodiazepines and non-benzodiazepine hypnotics within the data collection period. Such under and over exploitation of possibilities suggests inappropriate PRN prescriptions for some residents and should trigger the need for a medication review. For example, a regular dose may have been warranted. Additionally, this may indicate a trend of prescribing benzodiazepines and other sedating drugs in place of antipsychotics, given the drivers to reduce the prescription of the latter in people with dementia, which is of concern. In addition to antipsychotics and benzodiazepines, other medicines that were prescribed would be deemed potentially inappropriate, particularly in people with dementia. For example, tricyclic antidepressants, paroxetine and fluoxetine [39, 40]. Future research should explore this as there are clear guidelines regarding the long-term use of benzodiazepines within this population [43]. This is due to issues around dependency, toxicity, cognitive decline, falls and increased sensitivity towards side effects such as delirium [44, 45]. The needs of long-term care residents should be regularly reviewed to ensure only required medicines are prescribed and administered and that PRN prescriptions remain appropriate [6]. This is particularly important for those with dementia, as individuals with more severe cognitive impairment were less likely to receive pain relief on a PRN basis [20, 31] possibly as they struggle to communicate their symptoms [23], or expressions of pain through behaviours such as agitation are attributed as a symptom of dementia [46]. However, in our study, we found that there was a weak but significant relationship between number of PRN prescriptions and scores on an agitation measure. Further research is needed to understand this relationship and how well understood the needs of individuals experiencing agitation are.

Previous research suggests the main predictor of PRN medication is often the care home in which the resident lives [11]. We found the likelihood of being prescribed PRN medicines did not differ by care home. This could be because PRN prescriptions for some categories (i.e. antipsychotics) were too small to generate significant differences, rather than a lack of differences.

Our research implies that PRN use is lower than anticipated, but the current research base does not allow us to state, with confidence, that this is a genuine shift in expected prescription and administration. Future research should seek to collate the datasets from several large-scale clinical trials that have recently been completed in the UK, to establish whether this pattern of lower prescribing is seen more widely, before establishing whether a general trend towards lower PRN prescriptions for people with dementia is being seen generally.

### Strengths and limitations

This study presented the first examination of PRN medication prescription and administration within UK care homes for people with dementia. Whilst the data are cross-sectional, they provide an overview of current prescribing practices. Although, we do not know whether as dementia progresses, individuals are more likely to receive prescriptions for PRN medicines. Existing evidence suggests that as residents spend longer in care homes, their likelihood of receiving a PRN prescription increases [21]. Future work should seek to examine changes in prescriptions and administrations over time and their association with harms in care homes, such as falls and hospitalisations. Our data only included those falls resulting in hospitalisation. We are unlikely to have captured their true extent, and so analysing this data would have been inappropriate. Furthermore, whilst we have data on frequency of administrations, we do not have dosage or reason for prescription, which limited interpretation of the appropriateness of prescriptions. Heterogeneous data collection and research questions mean drawing comparisons between our findings and those of similar existing studies may be inappropriate.

## Conclusion

For the first time in the UK, this study presented the first evidence of the prescription and administration of PRN medicines for neuropsychiatric symptoms and pain management for people living with dementia. Generally, low levels of prescriptions and even lower levels of administrations were observed for the management of neuropsychiatric symptoms. This could be viewed positively as non-pharmacological treatments for dementia are being encouraged. However, the variation seen in PRN administrations suggests more research is needed to understand UK practices. The prescribing of potentially inappropriate medicines remains a problem in long-term care and pharmacists have a key role in reducing inappropriate polypharmacy by undertaking medication reviews that consider both regular and PRN medicines.
